# Creating Reproductive Health Behavioral Profiles for Women of Reproductive Age in Niger Using Cross-Sectional Survey Data: A Latent Class Analysis

**DOI:** 10.3389/ijph.2023.1605247

**Published:** 2023-01-24

**Authors:** Leanne Dougherty, Nicole Bellows, Chaibou Dadi

**Affiliations:** ^1^ Population Council, New York, NY, United States; ^2^ Avenir Health, Glasonbury, CT, United States; ^3^ Conception Etudes Suivi Evaluation Appuis Formation, Niamey, Niger

**Keywords:** maternal health, reproductive health, segmentation, Niger, health profiles

## Abstract

**Objectives:** To identify health behavioral profiles for women of reproductive age in Niger.

**Methods:** We interviewed married women of reproductive age in Niger in April 2021 (N = 2,709). Latent class analysis based on sociodemographic and behavioral determinants was used to identify classes of women related to use of antenatal care, facility delivery, and modern family planning (FP) use.

**Results:** We found similar classes between the use of antenatal care and facility-based delivery classes with the first class composed of less educated and poor women with weaker behavioral determinants while the second class was more educated and had stronger behavioral determinants. In the facility-based delivery class was the presence of a third class that was poor and uneducated with low levels of knowledge and social norms, but in contrast had much higher levels of positive attitudes, self-efficacy, and partner communication than the first class. A fourth class of younger, more educated women with strong behavioral determinants emerged related to FP.

**Conclusion:** The application of empirical subgrouping analysis permits an informed approach to targeted interventions and resource allocation for optimizing maternal and reproductive health.

## Introduction

High maternal and child mortality rates continue to burden Francophone West Africa, a sub-region characterized by high fertility and low utilization of reproductive and maternal health services. Niger has the highest fertility rate in the world at 6.8 births per woman as well as a high maternal mortality rate (509 maternal deaths per 100,000 live births) and high child mortality rate (80 child deaths per 1,000 live births) [1–3]. Contributing to these poor health indicators are low levels of utilization of antenatal care (ANC), facility-based births, and family planning (FP), where, in Niger, only 33% of women who had given birth in the past 5 years have attended at least four ANC visits, 60% of births occurred at a health facility, according to the most recent Demographic Health Survey [4] and only 18% of married women were using modern FP [5].

Improving the health-seeking behaviors and subsequent utilization of these critical health services can yield much needed gains in maternal and newborn health in Niger. Adequate ANC allows for the diagnosis and treatment of pregnancy-related complications, and facility-based deliveries enable the use of life-saving procedures when delivery complications occur. As such, both ANC and facility-based deliveries are associated with reduced infant mortality [6, 7]. Additionally, an increase in the use of modern FP can reduce maternal mortality by reducing the risk of unsafe abortion, delaying the first pregnancy among young women with premature pelvic development, and decreasing risks associated with high parity and closely spaced pregnancies [8].

Understanding the specific determinants and barriers to health-seeking for reproductive and maternal health services are critical to developing programs that can best address the needs of the community. There are several factors that can influence health-seeking behaviors for these services, including socioeconomic variables (e.g., education, wealth), as well as determinants outlined in the theory of planned behavior which include knowledge, perceived self-efficacy, attitudes, and perceived community gender and other social norms. One systematic review on the determinants of ANC utilization in sub-Saharan Africa found that in addition to socioeconomic factors, knowledge of the dangers of pregnancy and the number of recommended ANC visits, as well as positive attitudes toward ANC utilization, were key predictors of adequate ANC use, particularly for those in rural areas where knowledge levels are lower [9]. In Niger, Begum et al. found that women who received advice from their husbands about ANC were more likely to attend ANC services [10]. Similarly, a review on the determinants of facility-based births in sub-Saharan Africa found that knowledge of the risks of pregnancy and delivery and positive attitudes toward a facility-based birth, as well as women’s empowerment and autonomy, were important predictors of facility-based deliveries [11]. For use of modern FP, Blackstone et al.’s review of factors influencing contraceptive use in sub-Saharan Africa found that male disapproval and social norms surrounding fertility were barriers to modern FP use, while partner communication is positively associated with modern FP use [12].

In Niger, a handful of studies have examined the intermediate determinants of health-seeking behavior for reproductive and maternal health services. For utilization of ANC services, one qualitative study found that while all focus group members believed that ANC was important to their health, fewer than half knew the recommended number of visits [13], while another quantitative study found that wealth status (high vs. low wealth) was associated with adequate ANC use and facility-based birth in Niger, despite complete fee exemption policies for all women [14]. For use of contraception, a 2015 study found that social norms were an important factor for women with no formal schooling, whereas an individual’s positive attitudes toward contraception were more of a driving factor for those with formal schooling [15]. Challa et al. found that spousal communication among adolescent wives and their husbands in Niger was positively associated with contraceptive use [16]. Dalglish et al. used tools informed from marketing research to identify five sub-groups of women in Niger with distinct FP needs, attitudes, and patterns of use, indicating that demand creation may be more effective in increasing FP demand if interventions address the needs, values, and underlying beliefs of each sub-group [17].

To address the health-seeking behavior and health services utilization challenges experienced in Niger, the US Agency for International Development’s (USAID) Resilience in the Sahel Enhanced (RISE) initiative was initially conceived in 2012 as a response to a pattern of recurring severe droughts and other stressors undermining health development in the region [18]. Focused on the chronically vulnerable populations in Niger and Burkina Faso, the RISE program has strengthened the capacity of state institutions and local governance, increased sustainable economic wellbeing, and improved health and nutrition [18]. The subsequent RISE II program (2018–2023) builds on these accomplishments and has expanded to investments aimed at improving priority behaviors in maternal, newborn and child health (MNCH), FP, and water, sanitation, and hygiene (WASH) through an integrated social and behavior change (SBC) strategy [18]. By integrating SBC programs across sectors and health areas, RISE II leverages resources to address common underlying barriers to health-seeking behaviors, such as related attitudes and norms [19].

Effective SBC interventions incorporate audience segmentation which refers to the practice of dividing an audience into subgroups based on demographic, psychographic, and/or behavioral factors to develop tailored SBC approaches that are most likely to resonate with the audience subgroup [20]. Latent class analysis (LCA) identifies a set of subgroups based on patterns of responses across study participants in survey data and is increasingly being applied in social and behavioral science research to understand risk profiles in the health field [21]. LCA allows us to move beyond singularly focusing on one characteristic at a time (e.g., age) and instead finds relationships within the data that provide a more nuanced understanding of audience profiles using multiple characteristics at the same time. Several studies focused on HIV prevention have generated risk behavioral profiles to enable strategic, data driven programming [22–24], but application of audience segmentation profiles beyond sociodemographic characteristics in reproductive and maternal health programs is limited [25]. The objective of this study is to first develop health behavior profiles based on demographic and intermediate behavioral determinants influencing health-seeking behaviors for ANC use, facility-based delivery, and modern FP use. Next, we assess correlations between health behavioral profiles and reproductive and maternal health service utilization, hypothesizing that weaker intermediate behavioral determinants will be associated with lower rates of health-seeking behavior.

## Methods

### Study Site

As part of the RISE II program, the SBC interventions aimed at improving health-seeking behaviors are led by three Resilience Food Security Agencies (RFSAs) working in the Maradi and Zinder regions of Niger, each operating within communes in their respective zones. In total, the RFSAs are working in 18 communes within five departments. We randomly selected two communes from each of the three RFSA intervention zones and two neighboring communes per intervention zone as comparison zones with similar sociodemographic characteristics, healthcare accessibility, and population density.

### Study Design and Sampling

The study team administered a cross-sectional survey in April 2021 which aims to serve as a baseline for a quantitative evaluation of the RISE II integrated SBC program. We applied a three-stage stratified sampling procedure. In the first stage, we randomly selected six intervention communes and six control communes (four in Zinder and two in Maradi region). In the second stage, we listed all enumeration areas (EA) identified in the 2012 General Census by commune in each of the randomly selected communes. We then used probability proportion to size to select EAs per commune starting at a random point and then systematically selecting areas using a fixed sampling interval.

In total, we sampled 40 EAs for each group, stratified by commune. In the third stage, we enumerated all households in each of the randomly selected EAs with eligible women (married women between 15 and 49 years of age). From households with eligible women, we randomly selected 34 households per EA to account for a 10% non-response rate and interviewed 30 ever married women between 15 and 49 years of age. Due to lower than anticipated non-response rate (less than 1%), we arrived at a final sample size of 2,709. This sample size is based on a minimum detectable difference for evaluation purposes of 6–11 percentage points in the priority indicators (four or more antenatal care visits, facility delivery and modern contraceptive use) between study groups, with 80% power to detect a difference and alpha of 0.05.

### Measures

The principal outcomes of the survey were use of four or more ANC visits, delivering at a health facility among women who have had a child in the 5 years preceding the survey, and modern contraceptive use among all non-pregnant women participating in the survey. To inform the reproductive and maternal health behavioral profiles, we collected additional information on sociodemographic information including household assets (to compute a wealth index using the Equity Tool methodology) [26], age, education, marital status (monogamous or polygamous), as well as intermediate behavioral determinants related to knowledge, attitudes, self-efficacy, social norms, and partner communication related to the priority health outcomes. A summary of measures is described in Table 1.

**TABLE 1:** Reproductive and maternal health demographic and behavioral determinant measures (Maradi and Zinder, Niger, 2021).

Variable	At least 4 ANC visits for most recent birth in last 5 years	Facility-based delivery for most recent birth in last 5 years	Currently using a modern method of contraception
Sociodemographics			
Location	Categorical, three categories. Based on study communes and stratified by RISE II implementing partner intervention areas		
Age	Categorical, five categories. 15–24, 25–29, 30–34, 35–39, 40–49 based on question “How old are you in complete years?”		
Education	Binary, defined as no education or any education, based on the question “Have you gone to school?”		
Wealth	Categorical, household wealth tertiles constructed using Equity tool methods for Niger		
Marital	Binary, defined as polygamous/divorced/widowed/separated or monogamous based on questions “Are you currently married, living with a man like you are married, widowed, divorced, or separated?”		
Intermediate behavioral determinants			
Knowledge	Binary, reported 4 or more based on the question “How many ANC checkups should a woman get for her health and her baby’s health?”	Binary, reported a woman should give birth at a health facility/hospital based on the question “Where should a woman give birth?”	Binary, reported heard of at least 3 different FP methods, based on the question “Have you ever heard of [prompt method]?”
Attitudes	Ordinal, with 3 categories (agree, neutral, disagree) based on the question “To what extent are you in agreement with the statement: Pregnant women only need antenatal care if they are sick” (coded in reverse direction)	Ordinal, with 3 categories (agree, neutral, disagree) based on the question “To what extent are you in agreement with the statement: The health facility is the best place to deliver a baby”	Ordinal, with 3 categories (agree, neutral, disagree) based on the question “To what extent are you in agreement with the statement: It is acceptable for a couple to use methods such as condoms, the pill, or injectables to delay or avoid pregnancy”
Norms	Binary, reported 4 or more based on the question “How many ANC checkups do most women in this community receive?”	Binary, reported a woman should give birth at a health facility/hospital based on the question “Where do most women in this community give birth?”	Ordinal, with 3 categories (agree, neutral, disagree) based on the question “To what extent are you in agreement with the statement: Members of this community agree if a woman uses contraception”
Self-efficacy	Ordinal, with 3 categories (very difficult, somewhat difficult, not difficult) based on the question “Tell me how difficult you find the following action to perform: Get to a health facility for ANC”	Ordinal, with 3 categories (very difficult, somewhat difficult, not difficult) based on the question “Tell me how difficult you find the following actions to perform: Get to a health facility to give birth”	Ordinal, with 3 categories (agree, neutral, disagree) based on the question “To what extent are you in agreement with the statement: I know where to go to obtain contraception”
Partner communication	Ordinal, with 3 categories (agree, neutral, disagree) based on the question “To what extent are you in agreement with the statement: It is not at all difficult to start a conversation with husband/partner about attending antenatal care at a health facility”	Ordinal, with 3 categories (agree, neutral, disagree) based on the question “To what extent are you in agreement with the statement: It is not at all difficult to start a conversation with husband/partner about giving birth in a health facility”	Ordinal, with 3 categories (agree, neutral, disagree) based on the question “To what extent are you in agreement with the statement: I feel comfortable discussing family planning with my partner”

### Latent Class Analysis

For each of the three health-seeking behaviors in this study, ANC utilization, facility-based delivery, and use of modern FP, LCA is used to categorize the sample into classes, based on these measures of demographic and intermediate determinants influencing health-seeking behavior, which we then qualitatively explored and defined. We included intermediate behavioral determinants in the LCA specific to the behavior. For example, for FP knowledge, we included “Has ever heard of at least three different FP methods”; and for FP attitude we included “Agree it is acceptable for a couple to use methods such as condoms, the pill, or injectables to delay or avoid pregnancy.” The LCA followed an iterative process that involved constructing a series of models and refining the variables included. We began fitting 1- to 4-class models. With each model, we assessed interpretability and overall model fit. Final model selection was based on identification and relative-fit statistics as well as interpretability. For interpretability, we considered whether the latent classes were segmented in patterns consistent with the literature, (e.g., more educated and wealthier classes had stronger intermediate behavioral determinants) and were distinct from each other. The final model yielded probabilities for class membership, or the likelihood that an individual is properly classified and assigned to the best fitting class, and, for each class, item response probabilities for each indicator. Item response probabilities allow for the comparison of these probabilities between classes to assess how distinct each class is from one another. All analyses were conducted in Stata v16 [27].

### Post-Estimation Analysis

For post-estimation analyses, each respondent was assigned to a class based on their highest posterior latent class probability, in other words the probability that the respondent is classified in a given class. We then assessed associations between class membership based on these demographic and intermediate behavior determinants and the three health-seeking behaviors: attendance at four or more ANC visits, facility-based delivery, and modern FP use using logistic regressions. The comparison zones were selected based on similar sociodemographic characteristics, healthcare accessibility, and population density with the intervention zones. We conducted chi-squared tests to assess differences between the two groups across sociodemographic and behavioral outcomes. We determined that the samples were not statistically different and therefore pooled intervention and comparison communes from this baseline into a single analytic sample and stratified the post-estimation analysis by RFSA geographic area. We did not adjust for demographic characteristics because those characteristics were included in the LCA models.

### Ethical Approvals

The Ministry of Public Health National Ethics Committee for Health Research in Niger provided approval for the study and consent forms (No. 017/2020/CNERS). The study also received approval from the Population Council Institutional Review Board in the United States (Protocol number 934). All participants in the study provided written informed consent for their participation and were not provided any incentives for their participation.

## Results

Descriptive statistics for the sample are shown in Table 2. The respondents are located evenly among the three RFSA zones and the median age of respondents is 30 (standard deviation (SD) 6.8). Most women are in monogamous marriages (63%) while the remaining 38% are either in polygamous marriages or they are divorced, widowed, or separated. A large majority of the respondents report having had no formal schooling (85%). Looking at the knowledge and attitudes variables, we see relatively high levels of knowledge and positive attitudes toward the three health services. Over 90% of respondents agreed that the health facility is the best place to deliver a baby and 74% agreed that it is acceptable to use FP. Similarly, we see high levels of reported self-efficacy, with 80% or more reporting that it is not difficult to get to a health facility for ANC or delivery care and a similar percentage stating they know where to go for contraception. When reflecting on the broader community, we see somewhat lower levels of agreement that most women in the community agree with or use these health services, with 64% reporting that women in their community have four or more ANC visits, 67% agreeing that most women give birth in a health facility, and 63% agreeing that community members agree with the use of contraception. In terms of partner communication, most agreed that they could start a conversation with their husband/partner about attending ANC (89%) or giving birth in a health facility (84%), while only 64% stated they felt comfortable discussing FP with their partner.

**TABLE 2:** Description of Resilience in the Sahel Enhanced II study sample among married women 15–49 years (N = 2,709) (Maradi and Zinder, Niger, 2021).

Variable	%
Demographics	
Location	
RFSA zone 1 (Maradi)	35.2
RFSA zone 2 (Zinder)	35.1
RFSA zone 3 (Zinder)	29.7
Age Median (SD)	30 (6.8)
15–24	16.6
25–29	21.0
30–34	28.9
35–39	19.4
40–49	14.1
Marital	
Polygamous/Divorced/Widowed/Separated	37.5
Monogamous	62.5
Education	
No schooling	85.4
Some schooling	14.6
Wealth tertial (wealth)	
Poorer	33.7
Middle	33.0
Richer	33.3
Knowledge	
Knows that a woman should have 4 or more ANC checkups for her health and her baby’s health	77.4
Knows that a woman should give birth at a health facility	83.1
Has ever heard of at least 3 different FP methods	77.7
Attitudes	
Disagree that pregnant women only need antenatal care if they are sick	76.2
Agree the health facility is the best place to deliver a baby	93.1
Agree it is acceptable for a couple to use methods such as condoms, the pill, or injectables to delay or avoid pregnancy	74.0
Norms	
Agree that most women in the community have 4 or more ANC visits	64.3
Agree that most women in the community give birth at a health facility	67.2
Agree that members of this community agree if a woman uses contraception	62.9
Self-efficacy	
Agree that it is not at all difficult to get to a health facility for antenatal care	88.3
Agree that it is not at all difficult to get to a health facility to give birth	79.5
Agree that they know where to go to obtain contraception	80.1
Partner communication	
Agree that it is not at all difficult to start a conversation with husband/partner about attending antenatal care at a health facility	88.5
Agree that it is not at all difficult to start a conversation with husband/partner about giving birth in a health facility	83.7
I feel comfortable discussing family planning with my partner	64.2
Behavioral outcomes	
4 + ANC visits among women who have had a child in the 5 years preceding the survey (N = 2,537)	53.6
Delivering at a health facility for most recent birth among women who have had a child in the 5 years preceding the survey (N = 2,537)	56.0
Modern contraceptive use among all non-pregnant women (N = 2,388)	26.2

The results of the LCA are presented in Tables 3, 4. When considering the health behavior of having had at least four ANC visits for their last pregnancy, two different classes emerged. Class 1, which represents 29% of the total sample was less educated and poorer with weaker intermediate behavioral determinants (Table 3). In contrast, the 71% of the sample in Class 2 were somewhat more educated (17% vs. 10% with any schooling) and had stronger intermediate behavioral determinants, especially with regards to knowledge and norms, as compared to Class 1.

**TABLE 3:** Antenatal care and facility-based delivery classes among the Resilience in the Sahel Enhanced II study sample of married women 15–49 years (N = 2,537) (Maradi and Zinder, Niger, 2021).

	ANC 4+		Facility-based delivery		
	Class 1: Weak determinants	Class 2: Strong determinants	Class 1 weak determinants	Class 2 mixed determinants	Class 3 strong determinants
Class membership probability	28.8	71.1	20.0	12.4	67.6
Item Response Probabilities					
Demographic characteristics					
Women’s age					
15–24	18.4	15.8	21.3	16.2	15.2
25–29	18.4	22.0	20.1	19.0	21.6
30–34	27.1	29.7	27.3	32.5	28.8
35–39	16.9	20.4	15.7	15.6	21.1
40–49	19.1	12.1	15.5	16.6	13.2
Wealth tertile					
Lowest	45.6	29.0	51.9	50.0	25.4
Middle	36.3	31.6	38.4	38.7	30.3
Highest	18.1	39.4	11.3	11.3	44.3
Any education (ref: no education)	9.5	16.7	8.3	4.5	18.3
Marital: Monogamous (ref: polygamous)	74.1	57.8	74.6	80.6	55.6
Intermediate behavioral determinants^a^					
Knowledge	29.6	97.6	47.8	48.6	100.0
Attitude	60.2	83.0	74.4	95.3	98.4
Norm	20.2	94.9	16.0	30.7	95.7
Self-efficacy	61.7	99.3	0.8	100.0	99.2
Partner communication	64.2	98.6	30.2	97.0	97.6

^a^ Intermediate behavioral determinants included in the LCA are specific to the behavior. For example, ANC: knowledge (knows that a woman should have 4 or more ANC checkups for her health and her baby’s health); attitude (Disagree that pregnant women only need antenatal care if they are sick), etc.

**TABLE 4:** Family planning classes among the Resilience in the Sahel Enhanced II study sample of married women 15–49 years (N = 2,388) (Maradi and Zinder, Niger, 2021).

	Family planning			
	Class 1 Weak determinants	Class 2 Internally motivated and socially challenged	Class 3 Strong determinants, older	Class 4 Strong determinants, younger
Class membership probability	21.6	7.0	50.8	20.6
Item Response Probabilities				
Demographic characteristics				
Women’s age				
15–24	19.7	23.4	0.1	51.6
25–20	19.6	22.3	17.2	31.1
30–34	28.6	25.8	38.0	8.2
35–39	13.2	16.8	27.8	6.0
40–49	18.9	11.6	16.9	3.0
Wealth tertile				
Lowest	43.8	26.5	29.2	36.7
Middle	41.3	20.8	34.6	24.2
Highest	14.8	52.7	36.1	39.1
Any education (ref: no education)	8.3	22.6	7.7	35.3
Marital: Monogamous (ref: polygamous)	74.0	49.2	54.0	75.7
Intermediate behavioral determinants^a^				
Knowledge	38.5	87.3	89.4	90.6
Attitude	20.0	89.9	97.1	98.2
Norm	22.4	13.0	78.5	80.6
Self-efficacy	19.8	98.3	95.9	97.6
Partner communication	1.7	63.1	83.7	84.0

^a^ Intermediate behavioral determinants included in the LCA are specific to the behavior. For FP: knowledge (Has ever heard of at least 3 different FP methods); attitude (Agree it is acceptable for a couple to use methods such as condoms, the pill, or injectables to delay or avoid pregnancy), etc.

For facility-based delivery, three classes of respondents emerged. Class 1 represents 20% of the sample and consists of women that are younger, have low education, and are poorer. This group had weak intermediate behavioral determinants with low levels of knowledge, perceived social norms, perceived self-efficacy, and partner communication. Like Class 1, Class 2 (12% of sample) were more likely to be poor, uneducated, and have low levels of knowledge and perceived social norms conducive to desired behaviors; however, they are labeled “mixed determinants” due to having much higher levels of positive attitudes, self-efficacy, and partner communication than Class 1 but still low levels of knowledge and social norms. The remaining 68% of the sample were grouped into Class 3, where this group had higher levels of education, were wealthier, and had consistently strong intermediate behavioral determinants.

Table 4 shows the four classes that emerged when considering the use of modern FP. Class 1 represents 22% of the sample and includes respondents who are poorer, have low education, and weak behavioral determinants. Class 2 stands in contrast and while it only represents 7% of the sample, those in this group are wealthier and have higher education, and are labeled as “internally motivated and socially challenged” for having particularly strong attitudes and self-efficacy in spite of reporting low levels of social norms in support of FP. Class 3 represents over half the sample (51%) and has older women with low levels of education, but overall strong intermediate behavioral determinants, while Class 4 (21%) represents younger women (i.e., 51.6% are ages 15–24 in Class 4 compared to 23.4% in Class 2), with more education and also strong intermediate behavioral determinants.

For postestimation analyses, each respondent was assigned to a class. Class assignment diagnostics (Table 5) suggested a low chance of misclassification. The log likelihood shows higher values (closer to zero) with increasing degrees of freedom and the Akaike information criterion (AIC) and Bayesian information criterion (BIC) values were relatively lower indicating a better fit [21]. Entropy indicates how accurately the model defines classes. We found entropy values near or above 0.8, which is considered acceptable [28]. The probability of class membership and proportion assigned to each class overall were consistently distributed. Finally, the average posterior probabilities (AvePP) of class assignment ranged from 0.82 to 0.93, where ≥0.70 indicates high assignment accuracy [29].

**TABLE 5:** Model fit statistics and class assignment diagnostics for antenatal care, facility-based delivery and modern family planning classes among the Resilience in the Sahel Enhanced II study sample of married women 15–49 years (Maradi and Zinder, Niger, 2021).

Class	Log likelihood ratio	DF	AIC	BIC	Entropy	Probability of class membership (%)	Proportion assigned to class (%)	AvePP
ANC (4 + VISITS)								
Class 1	−16251.46	13	32528.926	32605.682	—	28.8	28.0	0.93
Class 2	−15262.06	27	30578.123	30737.540	0.816	71.1	72.0	0.96
Facility-based delivery								
Class 1	−16072.86	13	32171.719	32248.476	—	20.0	20.0	0.99
Class 2	−14456.73	27	28967.454	29126.871	0.89	12.4	11.0	0.88
Class 3	−14456.73	41	28639.488	28881.566	0.88	67.6	69.0	0.96
Modern FP use								
Class 1	−17849.31	13	35724.619	35801.375	—	21.6	21.5	0.92
Class 2	−16388.75	27	32831.510	32990.927	0.897	7.0	7.1	0.82
Class 3	−16293.56	41	32669.113	32911.191	0.839	50.8	58.1	0.84
Class 4	−16191.01	55	32492.014	32816.752	0.772	20.6	13.4	0.93

DF, degrees of freedom; AIC, akaike information criterion; BIC, Bayesian information criterion; AvePP, average posterior probabilities.

When examining the utilization of these three key health services by class membership, we see striking differences (Table 6). For ANC, only 25% in Class 1 obtained four or more ANC visits during their last pregnancy compared to 64% in Class 2% and 54% overall. Similarly, there is a strong association between class membership and facility-based births, with only 11% of those in Class 1 reporting having given birth in a facility, compared with 35% in Class 2% and 71% in Class 3. Finally, modern FP use was 23% overall, but also varied by class membership. Only 5% of Class 1 reported using modern FP, compared to 17% of the Class 2 “internally motivated and socially challenged.” Even higher levels of use were seen in Class 3 (29%) and Class 4 (30%) groups, which represented women with strong intermediate behavioral determinants of different age profiles.

**TABLE 6:** Factors between latent class membership and maternal health and family planning behavioral outcomes among the Resilience in the Sahel Enhanced II study sample of married women 15–49 years (Maradi and Zinder, Niger, 2021).

Behavioral outcome	Proportion	Odds ratio (CI)	Statistical Significance
ANC 4+ (n = 2,537)			
Full sample	0.54	n/a	
Class 1: Weak	0.25	Ref	
Class 2: Strong	0.64	5.49 (4.51–6.68)	(*p* < 0.000)
Facility-based delivery (*n* = 2,537)			
Full sample	0.56	n/a	
Class 1: Weak	0.11	Ref	
Class 2: Middle	0.35	4.19 (2.88–6.09)	(*p* < 0.000)
Class 3: Strong	0.71	19.3 (14.40–25.95)	(*p* < 0.000)
Modern FP use (*n* = 2,388)			
Full sample	0.26	n/a	
Class 1: Weak	0.05	Ref	
Class 2: Internally motivated and socially challenged	0.20	4.78 (2.77–8.24)	(*p* < 0.000)
Class 3: Strong, older	0.33	9.21 (6.16–13.79)	(*p* < 0.000)
Class 4: Strong, younger	0.34	9.63 (6.13–15.14)	(*p* < 0.000)

## Discussion

Audience segmentation is frequently used to identify population subgroups that have similar characteristics, needs, or behaviors to find more efficient ways to meet or generate demand for products and services [30]. To our knowledge, this is the first study to apply LCA to a cross-section of maternal and reproductive health behaviors to identify classes of women of reproductive age based on their sociodemographic and intermediate behavioral determinants. The study aims to further inform an integrated SBC program to increase use of maternal and reproductive health services by creating a more nuanced picture of which women are most vulnerable to poor reproductive and maternal health outcomes and which women may serve as potential conduits of information and behavior change motivation through peer-to-peer communication strategies [31].

There are three primary findings that came from this analysis. First, the descriptive statistics of the entire sample show fairly high levels of intermediate behavioral determinants among women in the intervention areas, with more than half of women responding positively to all the key questions around knowledge, attitudes, perceived enabling social norms, perceived self-efficacy, and partner communication. The two lowest reported intermediate behavioral determinants were agreement that “members of the community agree if a woman uses contraception,” and agreement that “I feel comfortable discussing FP with my partner.” Given that previous studies have found that male partners play an important role in determining fertility intentions [32] and contraceptive continuation [33], programs may find it beneficial to increase their focus on engaging the community and partners in conversations around FP [34].

Second, while the overall behavioral determinants are generally positive, it is clear from the LCA that there are high levels of variability among the respondents, with women clustering into groups of weak, mixed, and strong determinants. In this context, the advantage of LCA over other analytical approaches, such as logistic regression, is that it moves beyond individual variables to look at the intersection of multiple variables to identify groups of individuals that may benefit from a specific SBC intervention based on their shared characteristics. For example, for facility-based delivery, the “mixed determinants” (Class 2) group shows that while levels of education, knowledge, and perceived enabling social norms are low, similar to women in Class 1, the measures of attitudes, perceived self-efficacy, and partner communication are substantially higher and more comparable with Class 3. In this sense, one can think of women progressing along a continuum and focusing on the intermediate behavioral determinant that could facilitate that progression toward greater utilization of maternal health services. As with the Daglish et al. findings, which used a similar approach, the results from this study can be used to help guide the program focus and messaging based on the characteristics of the group and the levels of intermediate behavioral determinants within them [17]. For example, a recent study from Niger found that when health workers were trained on how to identify client segments and tailor counseling messages to clients based on a series of questions, clients expressed greater satisfaction based on the quality of care they received [35].

For use of modern FP, the two groups with the strongest intermediate behavioral determinants (Classes 3 and 4) are interesting in that Class 4 is younger and has more members with education and monogamous relationships; however, their intermediate behavioral determinants and FP use were nearly identical to those from the older and less educated Class 3 group. This suggests that both education and life experience can help build these intermediate behavioral determinants. In contrast, Class 1 members have weak intermediate behavioral determinants across the board, while Class 2 is a small but interesting segment of younger and wealthier women who see themselves outside the norms of their community, as evidenced by the weak perceived enabling social norms variable. The misalignment between attitudes and norms in Class 2 indicates that an SBC intervention might achieve change by unveiling the misperception that keeps people tied to the harmful norm [36].

While the compositions of the classes are interesting on their own, the third primary finding is that class membership is highly associated with the use of reproductive and maternal health services. This is not surprising given that group membership is a function of key demographics and the intermediate behavioral determinants, which have previously shown to be important predictors of health seeking behavior [9, 11, 12]. However, the differences between the weakest and strongest intermediate behavioral determinants groups are striking, particularly for facility-based deliveries and modern FP use, with high odds ratios in the highest class compared to the lowest class. Determining the extent that wealth versus the intermediate behavioral determinants versus the interaction of the two is the driving factor of these differences is a ripe area for future analyses. Findings from this study, which identify differences in classes beyond demographic data, further highlights the importance of collecting and analyzing intermediate behavioral determinant data to inform audience segmentation in SBC interventions.

### Limitations

As with any analysis, there are important limitations to acknowledge. First, this analysis uses cross-sectional data and thus cannot establish causation between group membership and health behaviors. Second, the overall high levels of intermediate behavioral determinants may be partially a function of social desirability bias, where some respondents may have responded more favorably than their actual opinions based on their expectations of what they think is the “correct” answer. Third, while this study examines partner communication, it does not factor in the importance of men’s actions or determinants, which have been shown to influence their wives’ health behaviors [37, 38].

Furthermore, while LCA is a rigorous statistical procedure, proper class assignment using this technique is not guaranteed and researchers can fall prey to “naming fallacy” where the class name does not accurately reflect class membership [39]. To avoid overly detailed names of the groups, we focused primarily on the weakness/strength of the intermediate behavioral determinants but recognize that group membership is complex and requires additional nuanced consideration by program planners and implementers.

### Conclusion

Despite these limitations, the findings from this analysis are useful for thinking about the characteristics and needs of the women in the RISE II intervention areas in Niger. The results presented here can be used for formulating SBC interventions in the program areas and targeting them to those with the weakest intermediate behavioral determinants and customizing the approach to best suit the group’s characteristics. Future research will explore how the SBC interventions impact these intermediate behavioral determinants and maternal health care and FP use.

## Abbreviations

AIC, akaike information criterion; AvePP, average posterior probability; ANC, antenatal care; BIC, Bayesian information criterion; CESAF, conception etudes suivi evaluation appuis formation; DF, degrees of freedom; FP, family planning; LCA, latent class analysis; MH, maternal health; RISE, resilience in the sahel enhanced; SBC, social and behavior change; SD, standard deviation; USAID, US agency for international development.
